# The expression of interleukin-1β in patients with chronic hepatitis B treated with pegylated-interferon-alpha combined with tenofovir disoproxil fumarate and monotherapy

**DOI:** 10.1186/s12876-023-02812-5

**Published:** 2023-05-19

**Authors:** Xiaoxia Hu, Haiying Luo, Guili Tan, Yadi Li, Bo Qin

**Affiliations:** Department of Infectious Diseases, Chongqing Key Laboratory of Infectious Diseases and Parasitic Diseases, the First Affiliated Hospital of Chongqing Medical University, No. 1 Youyi Road, Yuzhong District, Chongqing, 400016 China

**Keywords:** Pegylated interferon alpha, Tenofovir disoproxil fumarate, Chronic hepatitis B, Early response, Interleukin-1 beta

## Abstract

**Background:**

Anti-hepatitis B virus (HBV) treatment uses tenofovir disoproxil fumarate (TDF) along with Pegylated-interferon-alpha (Peg-IFN-α), which is more effective than TDF/Peg-IFN-α monotherapy. We have previously shown that interleukin-1beta (IL-1β) is related to the effectiveness of IFN-α treatment in chronic hepatitis B (CHB) patients. The aim was to investigate the expression of IL-1β in CHB patients treated with Peg-IFN-α combination with TDF and TDF/Peg-IFN-α monotherapy.

**Methods:**

Huh7 cells infected with HBV were stimulated by Peg-IFN-α and/or Tenofovir (TFV) for 24h. A single-center cohort study of prospective recruitment of CHB patients: untreated CHB (Group A), TDF combined with Peg-IFN-α therapy (Group B), Peg-IFN-α monotherapy (Group C), TDF monotherapy (Group D). Normal donors served as controls. The clinical datas and blood of patients were collected at 0, 12, and 24 weeks. According to the early response criteria, Group B and C were divided into two subgroups: the early response group (ERG) and the non-early response group (NERG). Stimulation of HBV-infected hepatoma cells with IL-1β to validate the antiviral activity of IL-1β. To test the blood sample, cell culture supernatant, and cell lysates and to assess the expression of IL-1β and HBV replication levels in various treatment protocols, Enzyme-Linked Immunosorbent Assay (ELISA) and quantitative reverse transcription polymerase chain reaction (qRT-PCR) were used. SPSS 26.0 and GraphPad Prism 8.0.2 software were used for statistical analysis. *P* values < 0.05 was considered to be statistically significant.

**Results:**

In vitro experiments, Peg-IFN-α plus TFV treatment group expressed higher IL-1β and inhibited HBV more effectively than monotherapy. Finally, 162 cases were enrolled for observation (Group A (n = 45), Group B (n = 46), Group C (n = 39), and Group D (n = 32)), and normal donors (n = 20) were enrolled for control. The early virological response rates of Group B, C, and D were 58.7%, 51.3%, and 31.2%. At 24 weeks, IL-1β in Group B(P = 0.007) and C(P = 0.034) showed higher than at 0 week. In Group B, the IL-1β showed an upward trend at 12w and 24w in the ERG. IL-1β significantly reduced HBV replication levels in hepatoma cells.

**Conclusion:**

The increased expression of IL-1β may enhance the efficacy of TDF combined with Peg-IFN-α therapy in achieving an early response for CHB patients.

## Background

Chronic hepatitis B (CHB) remains a significant public health issue worldwide, with approximately 250 million people worldwide currently infected [1, 2]. As such, the eradication of hepatitis B virus (HBV) is a considerable challenge [3]. At present, IFN-α and nucleos(t)ide analogues (NAs) are used as CHB treatment drugs [4–6]. NAs have been shown to reduce HBV-associated proteins and HBV-DNA in the serum of CHB patients[7, 8]. According to previous research, the use of tenofovir disoproxil fumarate (TDF) therapy significantly enhanced the cytotoxic activity of HBV specific CD8^+^ T lymphocytes [9]. Meanwhile, Pegylated-interferon-alpha (Peg-IFN-α) significantly enhanced serum clearance of HBsAg compared to NAs monotherapy [10, 11]. As is well known, IFN-α can induce hepatocytes to exert non-cytolytic antiviral effects by regulating gene expression and protein translation [12–15]. IFN-α also activates macrophages, natural killer cells, and T cells, with the ability to release cytokines, such as IL-6, IL-1β, and IL-4, thereby exhibiting antiviral effects at different stages of HBV replication [16]. However, the effectiveness of Peg-IFN-α and TDF is limited when used alone [3]. TDF and Peg-IFN-α have multiple modes of action, and combined application is a potential strategy that can exert complementary and synergistic effects. A randomized controlled trial found that for HBeAg-negative CHB, treatment with TDF plus Peg-IFN-α for 48 weeks is safe and has better effects than TDF monotherapy [17].

According to another study, in contrast to a non-response group, the interferon-response group had higher levels of IL-1β mRNA expression in IFN-α‐pre‐treated liver tissues [18]. Subsequently, IL-1β was discovered to be higher in CHB patients’ peripheral blood in the IFN-response group compared with the non-response group [19]. Notably, the pluripotent pro-inflammatory cytokine IL-1β has been demonstrated to inhibit HBV replication. Isorce [20] et al. stimulated hepatocytes with a variety of cytokines and found that IL-1β reduced HBV-DNA and showed higher anti-HBV activity than others. Additionally, a study indicated that patients with CHB had higher serum levels of IL-1β than patients who were not infected with HBV [21]. Hence, an assumption could be made that IL-1β plays a significant factor in the antiviral process of TDF combined with Peg-IFN-α.

In the present study, differences in IL-1β expression between healthy and untreated CHB were analyzed. At the same time, through cohort studies, the expression level and change trend of IL-1β and early virologic response rate in CHB patients treated with Peg-IFN-α combined with TDF and treated with Peg-IFN-α/TDF alone were observed. The expression level of IL-1β in different treatment regimens and the anti-HBV activity of IL-1β were also evaluated through in vitro experiments. The present study offers a significant foundation for an in-depth understanding of the pathogenesis of TDF combined with Peg-IFN-α in the treatment of CHB and the exploration of new treatments for CHB.

## Materials and methods

### Cell culture and plasmid transfection

Huh7 cells were donated by the Wei Xue research group and cultured with Dulbecco’s modified Eagle’s medium (DMEM) (GIBCO) supplemented with 1% penicillin/streptomycin and 10% fetal bovine serum (FBS) (Excell) at 37 °C in a 5% CO_2_ incubator. Huh7 cells transfected by HBV 1.3-mer WT were stimulated with 20µmol/L Tenofovir (TFV) (Meilunbio, China, Cat# MB1386-1, TFV is the main component of TDF antiviral) + 1000U/mL Peg-IFN-α-2b (Tebao, China), 1000U/mL Peg-IFN-α-2b, 20µmol/L TFV, or 0, 100, 200ng/mL IL-1β (BBI, China) for 24h. PBS was used as a control. HepG2.2.15 cells were stored in our research group and cultured by DMEM supplemented with 1% penicillin/streptomycin, 10% FBS, and 400ug/mL G418 (Meilunbio, China). HepG2.2.15 cells were stimulated with IL-1β for 48h in the absence of G418.

The HBV 1.3-mer WT plasmid was synthesized via JISSKANG (Shandong, China), and the corresponding empty plasmid was used as a negative control. Transfection was performed using Lipofectamine3000 (Invitrogen, USA) according to the manufacturer’s instructions.

### Study objects

The present study was an observational cohort study with the aim of determining early virological response rates and dynamic trends in IL-1β in different regimens for the treatment of CHB. The diagnostic criteria for CHB as defined by the 2019 guidelines for the prevention and treatment of CHB by the Chinese Society of Hepatology, is a chronic inflammatory disease of the liver caused by persistent infection with HBV. Timely optimization of individual treatment plans is integral to achieving a clinical cure for CHB and lowering the incidence of liver cirrhosis and liver cancer. Prospective recruitment was conducted by CHB patients aged 18 to 60 who attended the outpatient clinic of the Department of Infectious Diseases of the First Affiliated Hospital of Chongqing Medical University between June 2021 and September 2022. A total of 171 cases were enrolled for observation, and 9 cases were excluded. Finally, a total of 162 cases were included in the study and allocated to four groups: Group A (n = 45) - untreated CHB; Group B (n = 46) - CHB treated with a combination of TDF and Peg-IFN-α; Group C (n = 39) - CHB treated with Peg-IFN-α alone; Group D (n = 32) - CHB treated with TDF alone. In addition, 20 healthy donors were recruited for the control group. The study was conducted in accordance with the Declaration of Helsinki, and all patients provided informed consent and signed it before participating in the study.

(1) Criteria for Inclusion

The study included patients between the ages of 18–60 years who met the diagnostic criteria for CHB as defined by the 2019 China Hepatitis B Prevention and Treatment guidelines in China, with positive serum HBsAg and HBeAg, or negative HBeAg. The patients also tested positive or negative for HBV-DNA and provided consent for the use of Peg-IFN-α and TDF.

(2) Criteria for Exclusion

Patients were excluded from the study if they had other viral infections such as HIV, HEV, HCV, and other co-infections, end-stage manifestations of liver cirrhosis, liver failure, liver cancer, and other diseases, serious chronic diseases such as cardiovascular, urinary system, connective tissue, and blood system, experienced serious adverse events during the use of Peg-IFN-α, or did not provide informed consent.

(3) Criteria for early responding

An early response was defined as either a reduction in HBV-DNA > 2 log10 IU/mL or a rapid decrease in HBsAg during the 24-week treatment period (HBsAg < 200 IU/mL or a decrease > 1 log10 IU/mL at 12 or 24 weeks).

(4) Criteria for non-early responding

Non-response was defined as patients whose HBV-DNA had decreased by less than 2 log10 IU/mL at 24 weeks or HBsAg had decreased by less than 1 log10 IU/mL at 12 or 24 weeks.

### Treatment methods and observation indicators

#### Treatment methods

The patients in Group A had not received any anti-HBV therapy. The patients in Group B received 180ug/week of Peg-IFN-α-2b and 300 mg/day of TDF. Those in Group C were administered 180 µg/week of Peg-IFN-α-2b. Patients in Group D received 300 mg/day of TDF. Healthy donors served as control.

#### Observation indicators

The patients were observed at weeks 0, 12, and 24 (follow-up was stopped when patients experienced a serologic conversion of HBsAg). Blood samples and the following information were collected: (1) Each patient provided a 4ml sample of peripheral blood using an anticoagulant tube containing EDTA. The sample was then centrifuged at 3000 rpm for 20 min at 4 °C, and the plasma was collected. Peripheral blood mononuclear cells (PBMCs) were isolated and RNA was extracted. The plasma and RNA samples were stored at -80 °C; (2) Basic clinical information and epidemiological histories, such as the family history of hepatitis B, drug history and allergy history; (3) Blood tests, including routine blood (WBC and PLT) and liver function (ALT, AST, TBIL); (4) Virological indexes: serum HBV-DNA, HBsAg, HBeAg (HBV-DNA was expressed and calculated as 0 IU/mL when < 20 IU/mL, HBsAg was expressed and calculated as 25,000 IU/mL when ≥ 25,000 IU/mL; HBsAg were expressed and calculated as 0 IU/mL when < 0.05 IU/mL); (5) IL-1β concentration and IL-1β mRNA relative expression level: IL-1β concentration in plasma was detected using the ELISA kit, while IL-1β mRNA was measured by qRT-PCR in PBMCs; (6) Imaging data: color Doppler ultrasound of the upper abdomen/CT scan/MRI enhanced scan and liver transient elastography.

### Quantitative RT-PCR (qRT-PCR) assay

Total cellular RNA was extracted using Trizol plus (Takara, Bio). Reverse transcription was performed by using the primeScript^TM^RT reagent Kit (Takara, Cat# RR047A). Quantitative Real-time PCR reactions were performed on a system (Biorad) using SYBR^®^ Premix Ex Taq™ Kit (Takara, Cat# RR820A). The gene expression was normalized compared to β-actin and calculated utilizing the 2^−ΔΔCt^ method. The primers were as follows: β-actin, forward 5’-CAT GTA CGT TGC TAT CCA GGC-3’, reverse 5’-CTC CTT AAT GTC ACG CAC GAT-3’; IL-1β, forward 5’-ATG ATG GCT TAT TAC AGT GGC AA-3’, reverse 5’-CTC CTT AAT GTC ACG CAC GAT-3’; Pg RNA, forward 5’-GCC TTA GAG TCT CCT GAG CA -3’, reverse 5’- GAG GGA GTTC TTC TTC TAG G -3’; HBV RNA, forward 5’-ACC GAC CTT GAG GCA TAC TT-3’, reverse 5’- GCC TAC AGC CTC CTA GTA CA -3’.

### Enzyme-linked immunosorbent assay (ELISA)

HBsAg and HBeAg in the supernatant were measured using ELISA kits (Kehua Biotech, China). The protein expression levels of IL-1β were also measured using ELISA kits (J&L Biological, China).

### Isolation of peripheral blood mononuclear cells (PBMCs)

Ficoll-Paque Plus (Cytiva, Cat#17,144,003) was added to patient blood samples, and PBMCs were isolated by means of density gradient centrifugation.

### Statistical analysis

All variables were tested for normality by the Shapiro-Wilk test and histogram. Quantitative variables were expressed as mean ± standard deviation, medians (IQR), or counts (%). SPSS 26.0 and GraphPad Prism 8.0.2 software were used for statistical analysis and graphing of data. If the two sets of data followed a normal distribution, the t-test was used; otherwise, the Mann-Whitney U test was used. When comparing multiple sets of data, ANOVA or repeated measures ANOVA analysis was used if the data followed a normal distribution; otherwise, the Friedman M test was used. The Kruskal-Wallis test was used for three independent samples with nonnormal distribution use. The comparison of rates was tested with the Pearson χ^2^ test and χ^2^ test. A *P* value < 0.05 was considered to be statistically significant, as indicated by the following: *P < 0.05, **P < 0.01, ***P < 0.001, ****P < 0.0001.

## Results

### Peg-IFN-α in combination with Tenofovir showed stronger inhibition of HBV replication and promoted higher expression of IL-1β compared with monotherapy in cellular experiments

**Fig. 1:**
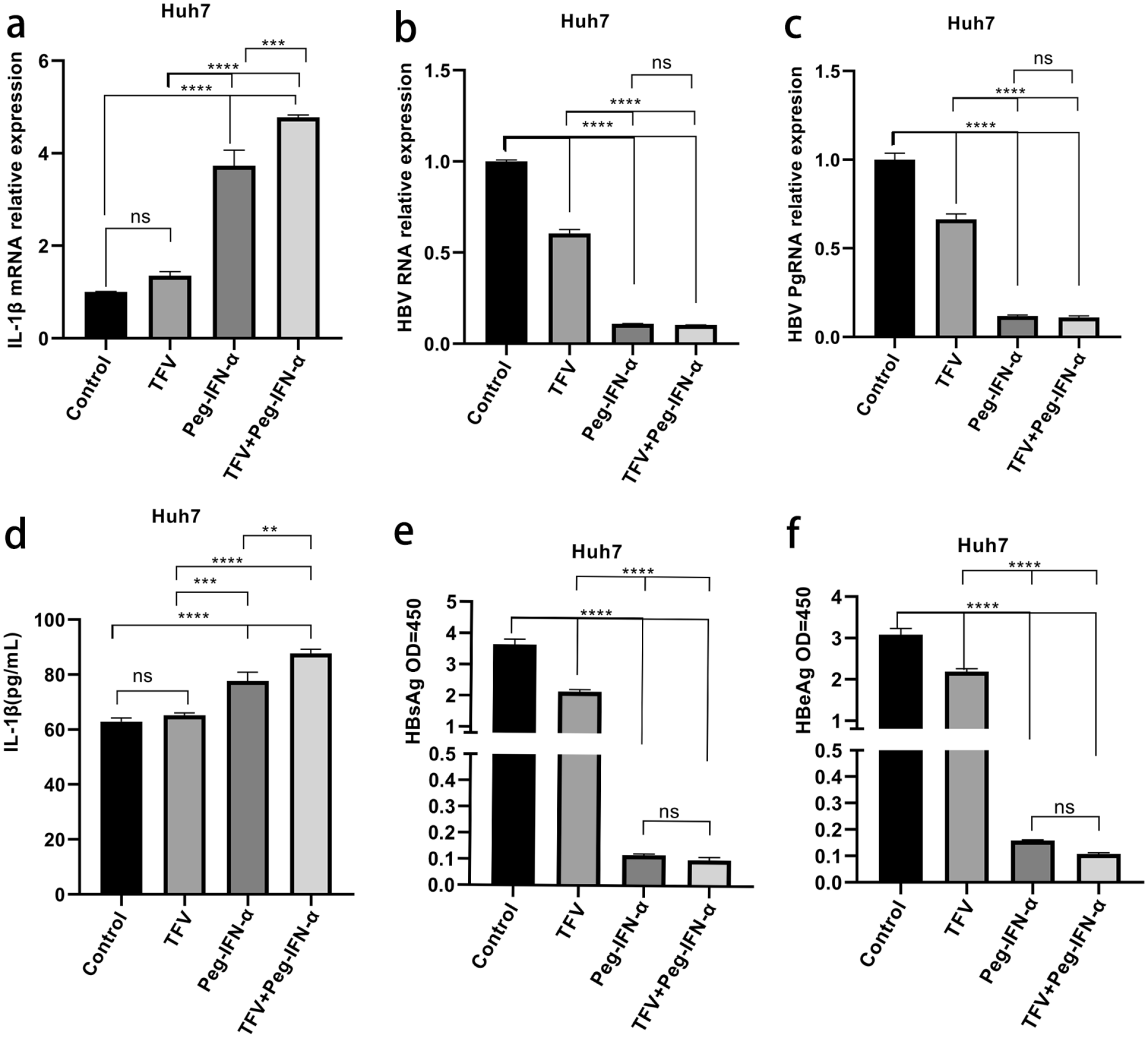
TFV + Peg-IFN-α promoted the expression of IL-1β and inhibited HBV replication: Huh7 cells were transfected with HBV 1.3-mer WT and treated with 20µmol/L TFV, 1000 U/mL Peg-IFN-α, and 20 µmol/L TFV + 1000 U/mL Peg-IFN-α for 24h **(a-c)** Cells were harvested and subjected to qRT-PCR. **(d-f)** The levels of supernatant IL-1β, HBsAg, and HBeAg were detected by ELISA. The results are presented as the means ± SD, *P < 0.05, **P < 0.01, ***P < 0.001, ****P < 0.0001

To investigate the expression of IL-1β in TFV in combination with Peg-IFN-α and TFV/Peg-IFN-α monotherapy,  Huh7 cells were transfected with HBV 1.3-mer WT and cultivated for 24h in the absence (PBS) or presence of TFV 20µmol/L, Peg-IFN-α 1000U/mL [19], and 20µmol/L TFV + 1000U/mL Peg-IFN-α. The expression levels of IL-1β mRNA, HBV RNA, and Pg RNA in cells were detected by means of qRT-PCR. ELISA was used to detect IL-1β, HBsAg, and HBeAg in cell culture supernatants. As shown in Fig. 1 (b, c, e, f), the TFV + Peg-IFN-α and Peg-IFN-α monotherapy significantly reduced HBV RNA, Pg RNA, HBsAg, and HBeAg compared with the TFV monotherapy (*P* < 0.0001). The expression levels of IL-1β mRNA and protein in the TFV+ Peg-IFN-α and Peg-IFN-α monotherapy groups were higher than those in the TFV monotherapy group. Notably, the TFV + Peg-IFN-α group expressed higher levels of IL-1β mRNA and protein than the Peg-IFN-α monotherapy group (Fig. 1(a, d)). In summary, the therapeutic effect of TFV + Peg-IFN-α was better than that of the Peg-IFN-α or TFV monotherapy groups and may enhance the antiviral ability by regulating the expression of IL-1β.

### Baseline characteristics of CHB patients

**Fig. 2:**
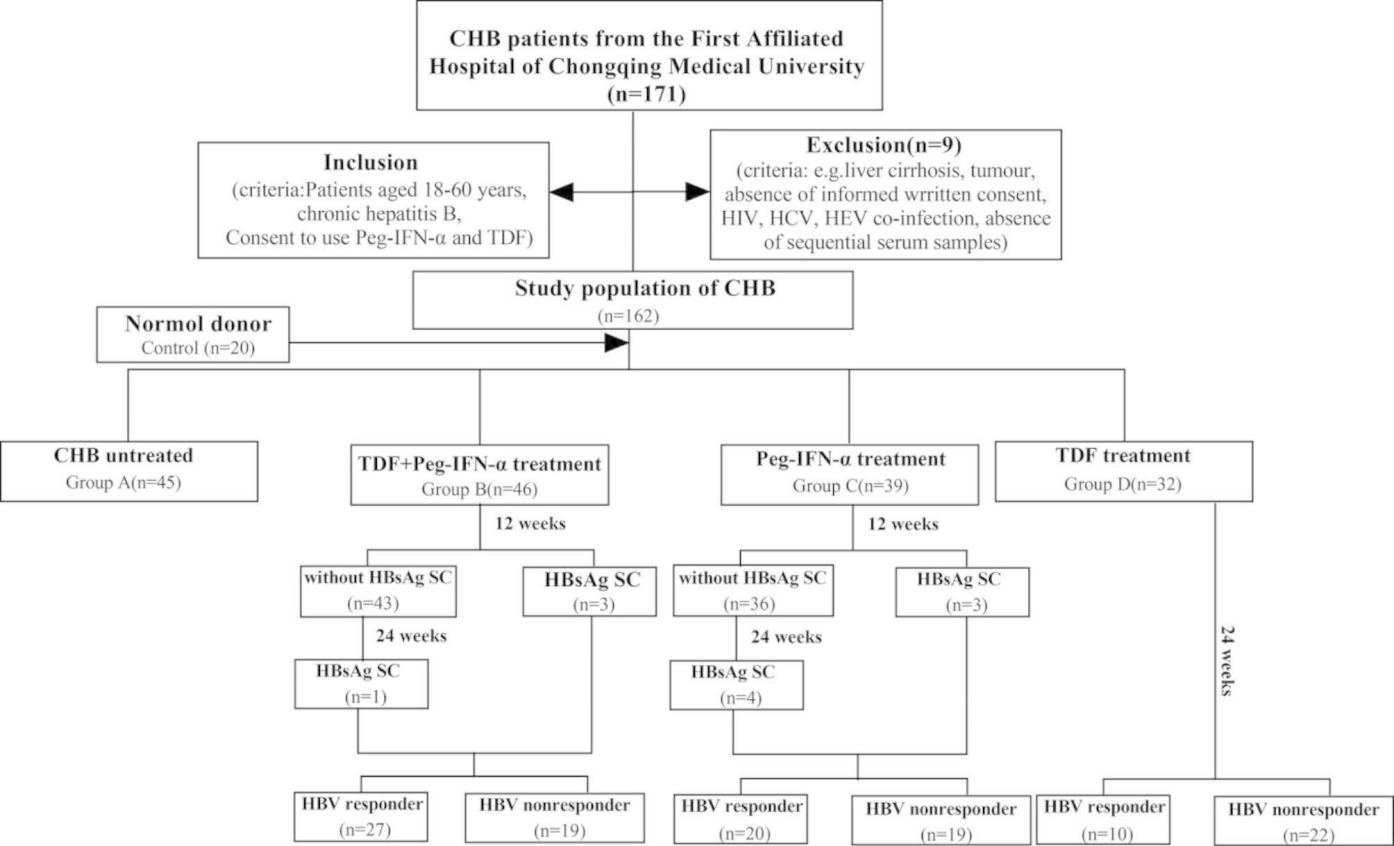
CHB patients enrolled in the follow-up process

In the present study, 171 CHB patients were recruited, and 9 cases were excluded owing to the absence of blood samples. Finally, 162 CHB were enrolled, with 45 cases in Group A, 46 cases in Group B, 39 cases in Group C, 32 cases in Group D, and 20 cases in the control group. The patients of Groups B, C, and D were followed for 24 weeks unless HBsAg serological conversion occurred. The follow-up procedure is shown in Fig. 2.

### The expression of IL-1β in patients with CHB was higher than in healthy donors

**Fig. 3:**
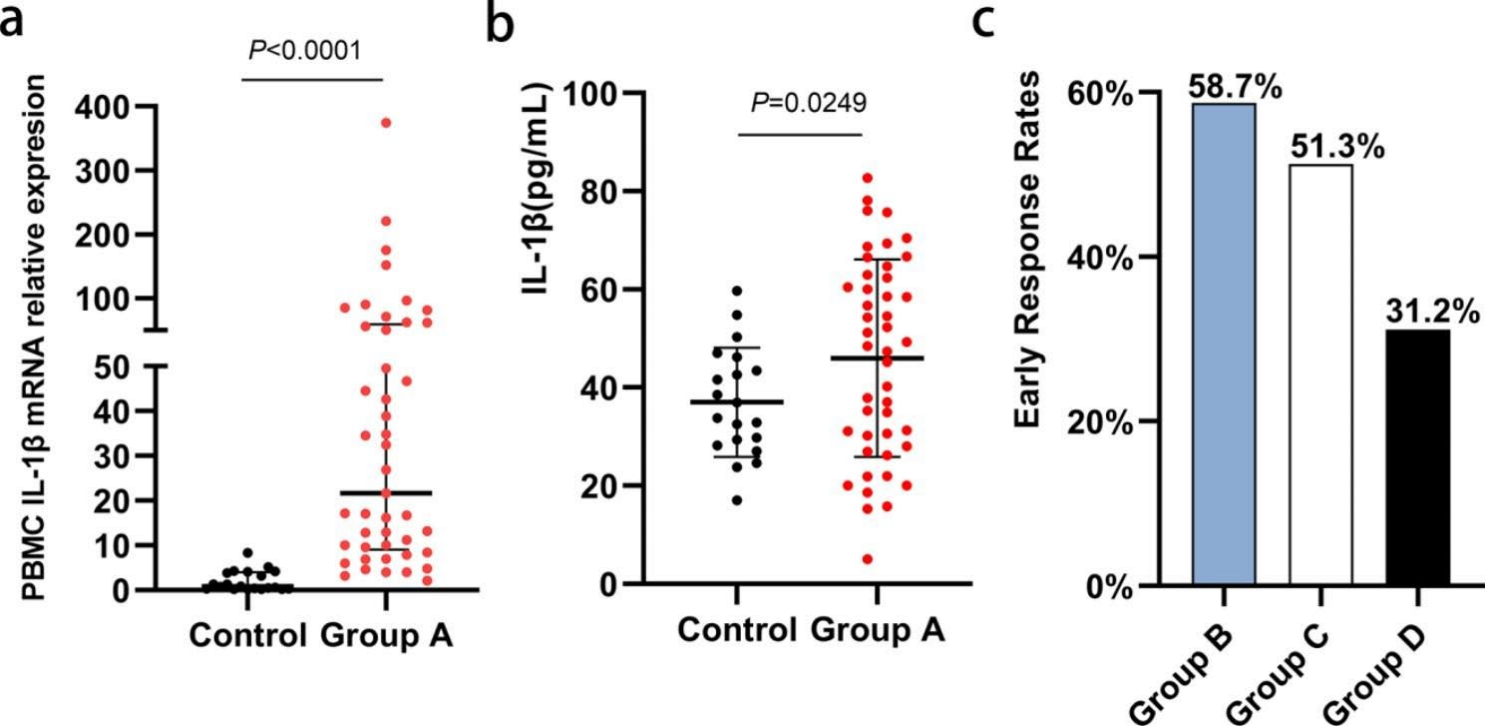
IL-1β expression and early response rates in different groups. **(a)** IL-1β mRNA in Group A and the control group was measured by means of qRT-PCR in PBMCs. **(b)** IL-1β in Group A and the control group was measured using ELISA in plasma. **(c)** Early virological response rates for Groups B, C, and D

Previously, a re-evaluation of previous Genome Oligo Microarray data collected by the present authors revealed significantly higher mRNA levels in IL-1β responders in IFN-α pre-treated liver tissue than in non-responders [19]. To further evaluate IL-1β expression in CHB patients, blood samples were collected from healthy individuals and untreated CHB patients, and IL-1β levels in plasma were measured using ELISA, while IL-1β mRNA in PBMCs was assessed using qRT-PCR. Compared with the control group, the mRNA and protein levels of IL-1β in Group A were significantly higher (Fig. 3(a-b)) (*P* < 0.05). There were no significant differences in age, gender, or family history of hepatitis B, ALT, AST, TBil, WBC, and PLT between Group A and the control group (*P* > 0.05), as shown in Table 1.

**Table 1:** Clinical characteristics of Groups A and control

Variables	Control (n = 20)	Group A (n = 45)	*P* value
Age (year)	36 ± 8.9	38.4 ± 10.4	0.378
Gender			
Male n (%)	8(40.0)	24(53.3)	0.321
Female n (%)	12(60.0)	21(46.7)	
Family history of hepatitis B			
Yes n (%)	6(30.0)	11(24.4)	0.638
No n (%)	14(70.0)	34(75.6)	
ALT (U/L)	27(21.5,35.8)	25(19,44.5)	0.915
AST (U/L)	26.5(18.8,32)	23(19,34)	0.96
TBIL (umol/L)	11.25(10.1,15.5)	10.9(8.4,14.7)	0.367
WBC (×10^9^/L)	5.8(5.2,7.1)	5.6(4.7,7.2)	0.271
PLT (×10^9^/L)	175.5(152.3,225)	195(166,218)	0.386
HBsAg IU/mL	0	1202.1(34.8,4861.3)	< 0.05
HBV-DNA IU/mL	0	796(0,407500)	< 0.05

Values are mean ± standard deviations(SD), medians(IQR), or counts(%), as appropriate. ALT: Alanine transaminase; AST: Aspartate transaminase; TBIL: total bilirubin; WBC: White Blood Cells; PLT: Platelets. HBsAg: Hepatitis B surface antigen; HBV-DNA: Hepatitis B virus deoxyribonucleic acid

### The early response rate and the increasing trend of IL-1β in the TDF combined with Peg-IFN-α group were higher than those in the Peg-IFN-α/TDF monotherapy group

At 0 week, there were no significant differences in age, gender, family history of hepatitis B, AST, ALT, TBIL, WBC, and PLT among patients in Groups B, C, and D(*P* > 0.05), as shown in Tables 2 and 3. At 12 and 24 weeks, compared with Group D, ALT and AST in Groups B and C were significantly increased, and WBC and PLT were significantly decreased (*P* < 0.05). In Groups B and C, the ALT and AST at 12 and 24 weeks were significantly higher than those at 0 week, and the WBC and PLT were significantly lower than those at 0 week (*P* < 0.05). In Group D, there were no significant differences in ALT, AST, DBIL, WBC, and PLT at 0, 12, and 24 weeks.

**Table 2:** Clinical characteristics of Groups B, C, and D at 0 week

Variables	Group B(n = 46)	Group C(n = 39)	Group D(n = 32)	*P* value
Age (year)	40(34,48.3)	39(29,46)	41.5(31,49.8)	0.499
Gender				
Male n (%)	26(56.5)	28(71.8)	17(53.1)	0.21
Female n (%)	20(43.5)	11(28.2)	15(46.9)	
Family history of hepatitis B				
Yes n (%)	15(32.6)	8(20.5)	12(37.5)	0.262
No n (%)	31(67.4)	31(79.5)	20(62.5)	

Values are expressed as medians (IQR) or counts (%) as appropriate

**Table 3:** Results of serum markers of HBV and biochemistry in treatment groups

Variables (Time)	Group B (n = 46)	Group C (n = 39)	Group D (n = 32)	*P* value
ALT(U/L)				
0 W	30 (20.5,45.3)^a^	21.2(16,32)	19 (14,27)^a^	< 0.05
12 W	53(30,83.3)^b^	57(35,70)^d^	19.5(15,26.8)	< 0.05
24 W	43.5(26.5,61.8)	50.5(35.3,65.8)^d^	19(16,24.8)	< 0.05
AST(U/L)				
0 W	23(19,33)	21(18,27)	23(16,26)	0.375
12 W	40.5(31,59.3)^b^	40(32,66)^d^	21(17.3,24.9)	< 0.05
24 W	35(26,50.3)^b^	46.5(32.3,57)^d^	20(18,24.8)	< 0.05
TBIL (umol/L)				
0 W	11.7(9.8,14.6)	10.9(7.6,15.9)	11.1(9.6,13.3)	0.672
12 W	9.9(7.8,13)^b^	9.9(8.2,13.1)	10.9(8.8,13.2)	0.605
24 W	8.5(6.8,10.4)^b^	9.9(8.3,11.8)^d^	10.9(8.5,13.0)	< 0.05
WBC (×10^9^/L)				
0 W	5.6(4.9,6.7)	5.6(4.7,6.4)	5.6(4.7,6.9)	1
12 W	3.1(2.6,4.4)^b^	3.0(2.6,3.7)^d^	5.7(4.7,7.0)	< 0.05
24 W	3.4(2.4,4.5)^b^	3.3(2.8,4.0)^d^	5.3(4.4,6.0)	< 0.05
PLT (×10^9^/L)				
0 W	185(157,229)	198(1177,244)	195(150.8,230.3)	0.149
12 W	108.5(90.5,148.8)^b^	111(86,135)^d^	195(150.3,232.8)	< 0.05
24 W	116.5(78.8,164)^b^	105.5(91.3,134.5)^d^	197.5(155.8,233.8)	< 0.05
HBsAg IU/mL				
0 W	1718.4(675.3,5945.1)	208.1(46.8,3451.6)^c^	2159.7(978.5,8360.6)^c^	< 0.05
12 W	1335.5(124.0,4081.7)^b^	92.9(3.49,1083.2)^e^	2254.6(1075.0,8734.4)	< 0.05
24 W	1001.3(10.9,2852.7)^b^	48.7(0.98,989.4)^d^	1959.5(748.9,7293.0)	< 0.05
HBV-DNA (-)				
0 W n (%)	26(56.5)	24(61.5)	19(59.4)	0.895
12 W n (%)	40(87.0)^b^	34(87.2)^d^	23(71.9)	0.151
24 W n (%)	40(87.0)^b^	32(82.1)^d^	26(56.5)	0.749
Early respond				
Yes n (%)	27(58.7)	20(51.3)	10(31.2)	0.054
No n (%)	19(41.3)	19(48.7)	22(68.8)	
HBsAg SC n (%)	4(8.7)	7(17.9)	0	

Values are expressed as medians (IQR) or counts (%) as appropriate

^a^ There was a significant difference in ALT between Groups B and D (*P *< 0.05);

^b^ The difference was statistically significant compared with week 0 in Group B (*P *< 0.05);

^c^ There was a significant difference in HBsAg between Groups C and D (*P* < 0.05);

^d^ The difference was statistically significant compared with 0 week in Group C (*P* < 0.05);

^e^ The difference was statistically significant compared with 24 weeks in Group C (*P* < 0.05);

Early response: early virological response. HBV DNA (-) < 20IU/ml; HBsAg SC: HBsAg seroconversion

After 24 weeks of follow-up, 4 patients in Group B achieved HBsAg SC, with a median (quartile) HBsAg level of 1001.3 (10.9, 2852.7) IU/ml at 24 weeks, which was significantly lower than the baseline level of 1718.4 (675.3, 5945.1) IU/ml (*P *< 0.05). In Group C, 7 patients achieved HBsAg SC, with a median (quartile) HBsAg level of 48.7 (0.98, 989.4) IU/ml at 24 weeks, which was significantly lower than the baseline level of 208.1 (46.8, 3451.6) IU/ml, but there was no significant difference from the level at 12 weeks (*P* > 0.05). None of the patients in Group D achieved HBsAg SC, and there was no significant difference in HBsAg levels between 0, 12, and 24 weeks (Table 3). In addition, the rate of HBV-DNA (-) attainment in Group B patients was significantly higher at 12 and 24 weeks than at 0 week (*P* < 0.05). Similarly, the proportion of patients in Group C who attained HBV-DNA (-) was significantly higher at 12 and 24 weeks than at 0 week (*P*<0.05). There was no significant difference in the proportion of patients that attained HBV-DNA (-) in Group D at 0, 12, and 24 weeks (*P* > 0.05). The early virological response rates of Groups B, C, and D were 58.7% (27/46), 51.3% (20/39), and 31.2% (10/32), respectively, there was no significant difference (Fig. 3c). However, the early virological response rate (EVRR) was higher in Group B (TDF combined with Peg-IFN-α) compared to the TDF/Peg-IFN-α monotherapy groups (Groups C and D).

**Fig. 4:**
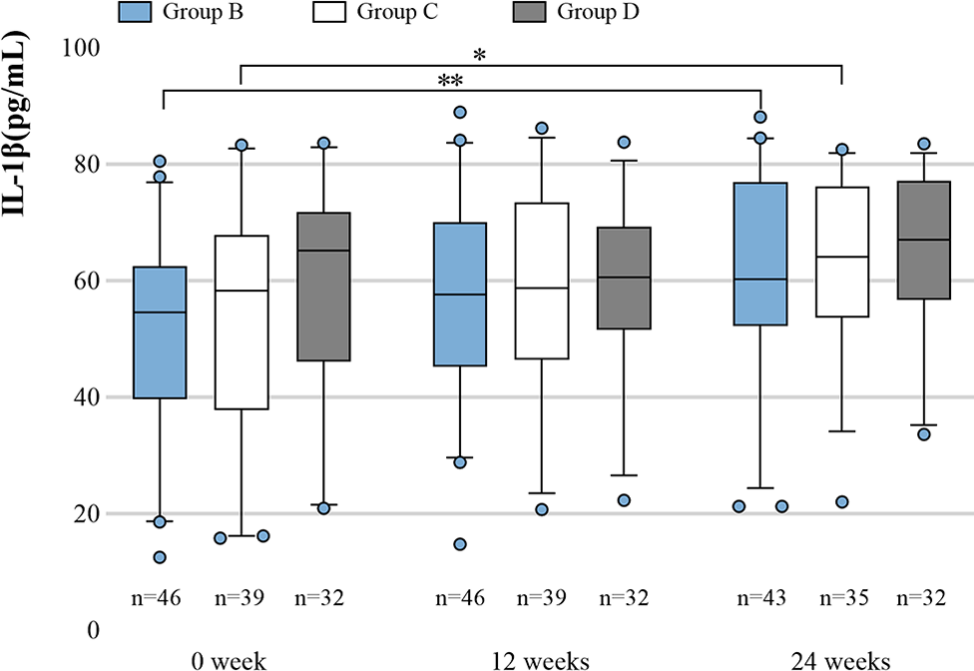
IL-1β expression in Groups B, C, and D. Plasmas were collected from CHB (including 46 patients in Group B, 39 patients in Group C, and 32 patients in Group D). ELISA was used to measure the levels of IL-1β in plasma. The results are presented as the median (IQR). * *P* < 0.05, **P < 0.01

The expression of IL-1β of Group B in the plasma of CHB patients at 24 weeks was significantly higher than that at 0 week (*P* = 0.007). Similarly, the IL-1β levels of plasma in Group C at 24 weeks were significantly higher than that at 0 week (*P* = 0.034). However, IL-1β in Group D did not differ significantly at 0, 12, and 24 weeks (*P* > 0.05). There were no statistically significant differences in IL-1β levels among Groups B, C, and D at 0, 12, and 24 weeks (Fig. 4).

### Subgroup analysis of Group B and C

**Fig. 5:**
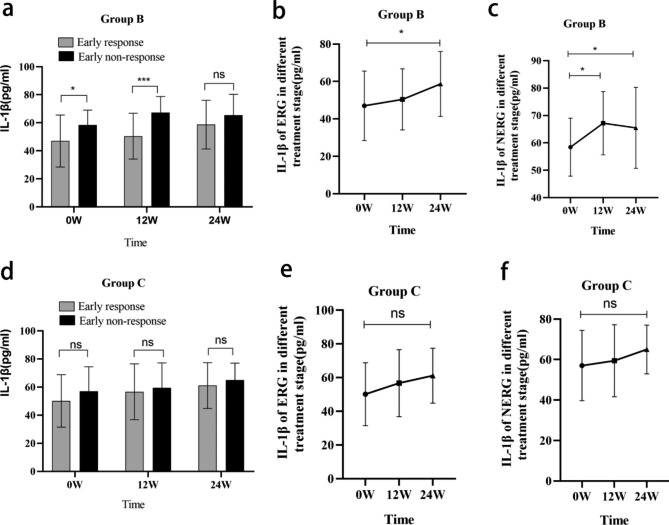
The expression of IL-1β in ERG and NERG of Groups B and C **(a-c).** The expression of IL-1β in ERG and NERG of Group B; **(d-f)** The expression of IL-1β in ERG and NERG of Group C;*P < 0.05, **P < 0.01, ***P < 0.001, ****P < 0.0001

Groups B and C were divided into ERG and NERG according to the criteria for early response. In Group B, IL-1β expression in the NERG was higher than in the ERG at 0w and 12w, respectively (Fig. 5a); the expression of IL-1β in ERG showed an increasing trend during treatment, and the expression of IL-1β at 24w was significantly higher than that at 0w (*P* < 0.05); the expression of IL-1β in the NERG was significantly increased at 12w and 24w compared with 0w (*P* < 0.05), but the expression of IL-1β at 24w demonstrated a downward trend compared with 12w. In Group C, there was no significant difference between the IL-1β expression response group and NERG at 0, 12, and 24 weeks (*P* > 0.05), respectively; the expression of IL-1β in ERG and NERG exhibited an increasing trend with the progress of treatment, but the difference was not statistically significant. (Fig. 5)

### IL-1β inhibited HBV transcription and HBV-related protein expression

**Fig. 6:**
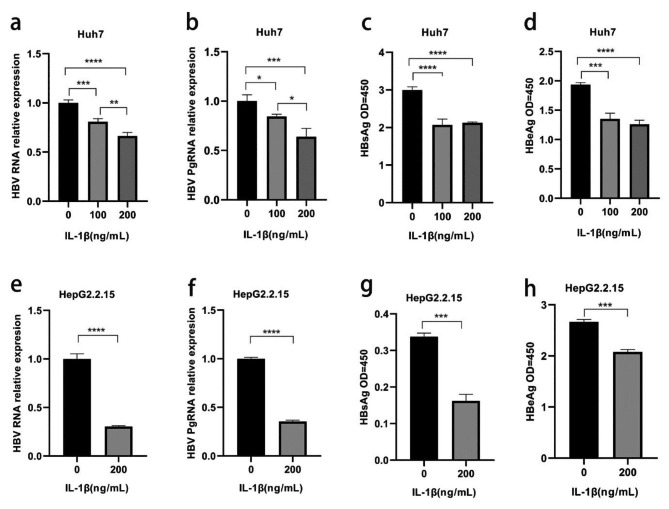
Anti-HBV activity of IL-1β in hepatoma cells. **(a-d)** Huh7 cells were transfected with HBV 1.3-mer WT plasmids and cultivated for 24h in the absence (PBS) or presence of IL-1β (100 and 200 ng/ml); **(e-h)** HepG2.2.15 cells were stimulated with 0 and 200ng/ml IL-1β for 48h; Cells were harvested and subjected to RT-qPCR. The HBsAg and HBeAg in the supernatant were analyzed using ELISA. *P < 0.05, **P < 0.01, ***P < 0.001, ****P < 0.0001

To investigate the potential antiviral activity of IL-1β, Huh7 cells were transfected with HBV 1.3mer WT and treated with IL-1β (0-200 ng/ml) for 24h. The treatment with IL-1β resulted in reduced levels of HBV RNA, Pg RNA, HBsAg, and HBeAg in Huh7 cells transfected with HBV (Fig. 6a-d). Additionally, IL-1β was found to decrease the level of HBV replication in HepG2.2.15 cells with stable HBV expression (Fig. 6e-h). The results confirm the anti-HBV activity of IL-1β in hepatocytes through in vitro experiments.

## Discussion

CHB can be controlled, but eliminating HBV remains a challenge. Currently, a functional cure is the optimal outcome. IFN-α and NAs are first-line drugs for CHB. TDF reduces liver cancer incidence with low resistance [22, 23], and has also been discovered to affect immunological function. The difficulty in treating HBV infections is primarily caused by cccDNA [11]. IFN-α has been the only treatment option until now for targeting persistent cccDNA within the hepatocyte nuclei of patients with chronic HBV infections through immunomodulation [24]. As such, through modifying the human immune response, the combination of TDF and Peg-IFN-α may be more beneficial than monotherapy in increasing host clearance of the HBV and re-establishing the body’s antiviral immune response function. In previous research by the present authors, IL-1β was found to be potentially associated with antiviral efficacy. In the present study, in vitro experiments were first conducted. As shown in Fig. 1, in vitro experiments were conducted to stimulate Huh7 cells infected with HBV using TFV, Peg-IFN-α, and TFV in combination with Peg-IFN-α. The findings revealed that the expression levels of IL-1β were higher in the TFV + Peg-IFN-α therapy group compared with those in the Peg-IFN-α/TFV monotherapy group. At the same time, treatments with TFV + Peg-IFN-α and Peg-IFN-α monotherapy, administered through Huh7 infection of the HBV model, were revealed to considerably suppress the levels of HBV RNA and Pg RNA. In conclusion, an assumption could be made that TDF + Peg-IFN-α promotes increased expression of IL-1β in patients with CHB, possibly related to the more effective inhibition of HBV replication.

In the present study, 45 untreated patients with CHB and 20 healthy individuals were enrolled, IL-1β expression in patients with CHB was found to be significantly higher than in healthy people. The aim of the present study was to evaluate the IL-1β expression in CHB patients who received TDF with Peg-IFN-α or monotherapy. A total of 117 CHB patients were categorized into Group B (TDF + Peg-IFN-α), Group C (Peg-IFN-α), and Group D (TDF). The results revealed that Groups B and C showed anomalies in WBC, PLT, AST, and ALT after initiating the treatment, possibly due to immunomodulatory and pro-inflammatory cytokine expression. Peg-IFN-α monotherapy and TDF with Peg-IFN-α were more effective in reducing HBsAg and HBV-DNA levels compared with TDF monotherapy. The EVRR was higher in Group B than in Groups C and D. According to the described data, TDF combined with Peg-IFN-α had better anti-HBV efficacy than Peg-IFN-α/TDF monotherapy. Subsequently, the changes in IL-1β expression during the follow-up period were assessed. The results indicate that the IL-1β levels in Groups B and C at 24 weeks were significantly higher than at baseline. Notably, the increase in IL-1β expression was more pronounced in the combination therapy group than in the Peg-IFN-α monotherapy group. Additionally, there was no significant difference in IL-1β levels in Group C at 0, 12, and 24 weeks, respectively. TDF combined with Peg-IFN-α treatment could promote increased expression of IL-1β in the ERG and the NERG, but the expression of IL-1β in the NERG decreased after 12 weeks, which may also be the reason for the early non-response in patients with CHB. No significant increase in IL-1β was observed in the treated Peg-IFN-α monotherapy group. Thus, the higher efficacy of TDF combined with Peg-IFN-α compared with monotherapy may be related to promoting the expression of IL-1β in patients. Undoubtedly, IFN-α can promote an increase in IL-1β [16]. As previously reported, HBV may inhibit the activation of inflammatory bodies and the production of IL-1β by inhibiting the activation of p-STAT1 and p-P65. As HBV is inhibited, the expression of IL-1β increases [19]. Therefore, the combined treatment of TDF and Peg-IFN-α can better inhibit the replication of HBV, while the inhibitory effect of HBV on inflammatory pathways is weakened, which is beneficial to the secretion of IL-1β. IL-1β assists TDF and Peg-IFNα to suppress HBV. Further, the results of the in vitro experiments indicate potent anti-HBV activity of IL-1β. A study found that IL-1β is a significant factor in controlling aggressive pathogens such as hepatitis B and C viruses by activating the expression of the specific immune gene [25, 26] and promoting lymphocyte recruitment to the primary site of infection [27, 28]. As such, the next step will be to investigate the IL-1β anti-HBV pathway.

The expression of IL-1β in Group B was comparatively lower than that in the early non-response group at 0w, possibly due to the high viral load, elevated expression of HBV-related proteins, and the presence of HBsAg > 3000IU/mL, HBeAg (+), and HBV-DNA (+) in patients with chronic hepatitis B (CHB), which may result in an elevated baseline IL-1β expression. The sequential treatment of TDF and Peg-IFN-α may have a better effect on the complete elimination of HBV in such patients.

## Conclusions

A better understanding of the mechanisms of Peg-IFN-α in the treatment of chronic hepatitis B in combination with TDF can facilitate the discovery of new treatment strategies to cure CHB. According to the present data, the combination therapy of Peg-IFN-α and TDF exhibited a higher virological response rate and increased IL-1β expression than Peg-IFN-α/TDF monotherapy. TDF combined with Peg IFN-α in treating CHB may enhance antiviral efficacy by increasing IL-1β expression. The described method has the potential to serve as a novel therapeutic approach for patients with CHB, particularly for non-responsive patients. The method allows for the assessment of treatment efficacy at an early stage and enables timely adjustments to individual treatment plans, potentially reducing the risk of complications. The present findings support the crucial role of TDF + Peg-IFN-α in controlling and eliminating HBV and shed light on how the hepatitis B virus interacts with the innate immune system. Despite the significant advantages, the present study needs to be validated in the context of a larger potential patient population and extended follow-up.

## Data Availability

The datasets generated and/or analysed during the current study are not publicly available due to protect the privacy of the patients, but are available from the corresponding author on reasonable request.

## Abbreviations

NAs: Nucleos(t)ide analogues
Peg-IFN-α: Pegylated-interferon-α
HBV: Hepatitis B Virus
CHB: Chronic hepatitis B
IL-1β: Interleukin-1beta
TDF: Tenofovir disoproxil fumarate
ALT: Alanine transaminase
AST: Aspartate transaminase
TBIL: Total bilirubin
WBC: White Blood Cells
PLT: Platelets
HBsAg: Hepatitis B surface antigen
HBV DNA: Hepatitis B virus deoxyribonucleic acid
